# Supplementary material for: Circadian Alterations in Brain Metabolism Linked to Cognitive Deficits During Hepatic Ischemia-Reperfusion Injury Using [1H-13C]-NMR Metabolomics
Source: Biomedicines. 2024 Nov 6;12(11):2536. doi: 10.3390/biomedicines12112536 (PMC11592224; doi:10.3390/biomedicines12112536)
Supplement: Supplementary file 1 [file biomedicines-12-02536-s001.zip › biomedicines-3246061-supplementary.pdf]

Supplementary Table S1.The metabolite enrichment levels in the CTR group at ZT0 and ZT12 with [2-^{13}C]-acetate infusion.

Metabolites	T value	P value	Mean difference	Standard error difference	CI for the difference	
					lower limit	upper limit
Lac_3	1.15	0.28	0.08	0.07	-0.07866	0.24066
Ala_3	-0.22	0.83	-0.14	0.62	-1.54169	1.26769
$GABA_3$	0.68	0.51	0.13	0.19	-0.30234	0.56167
NAA_3	-0.22	0.83	-0.02	0.10	-0.25835	0.21169
Glx_3	1.63	0.14	0.09	0.06	-0.03641	0.22575
$GABA_2$	0.71	0.50	0.16	0.22	-0.34771	0.66371
Glu_4	2.31	**0.05**	0.18	0.08	0.00399	0.36468
Gln_4	-0.29	0.78	-0.15	0.51	-1.31392	1.01125
Asp_3	-0.40	0.69	-0.03	0.08	-0.20203	0.1407
$GABA_4$	1.25	0.24	0.27	0.22	-0.21961	0.76027
Creatine	-0.42	0.69	-0.01	0.02	-0.05988	0.04121
Tau_1	-2.11	0.06	-0.03	0.02	-0.0718	0.00247
Myo_5	-0.74	0.48	-0.00	0.01	-0.01625	0.00825
Tau_2	-0.48	0.64	-0.01	0.02	-0.04783	0.03117
Myo_3	0.14	0.89	0.00	0.03	-0.06898	0.07831
Myo_4	-0.34	0.74	-0.01	0.02	-0.04074	0.03007
Glx_2	0.71	0.50	0.05	0.07	-0.11481	0.21948

Abbreviations:LactateC$_3$(Lac_3); AlanineC$_3$($AlaC_3$); N-acetylaspartateC$_3$(NAA_3); γ-aminobutyric acidC$_2$($GABA_2$); γ-aminobutyric acidC$_3$($GABA_3$); γ-aminobutyric acidC$_4$($GABA_4$); GlutamateC$_4$(Glu_4); GlutamineC$_4$(Gln_4); Glu_2+Gln_2(Glx_2); Glu_3+Gln_3(Glx_3); Aspartic acidC$_3$(Asp_3); TaurineC$_1$(Tau_1); TaurineC$_2$(Tau_2); Myo-InositolC$_3$(Myo_3); Myo-InositolC$_4$(Myo_4); Myo-InositolC$_5$(Myo_5). Bold indicated statistical significance.

Supplementary Table S2. The metabolite enrichment levels in the CTR and HIRI groups at ZT0 with [2-^{13}C]-acetate infusion.

Metabolites	T value	P value	Mean difference	Standard error difference	CI for the difference	
					lower limit	upper limit
Lac_3	0.99	0.34	0.06	0.06	-0.07461	0.19461
Ala_3	0.36	0.73	0.28	0.77	-1.43182	1.98515
$GABA_3$	1.20	0.26	0.20	0.17	-0.17505	0.58172
NAA_3	0.81	0.44	0.04	0.05	-0.07288	0.15621
Glx_3	1.04	0.32	0.06	0.06	-0.06983	0.19316
$GABA_2$	1.03	0.32	0.22	0.22	-0.25775	0.70441
Glu_4	3.41	**0.01**	0.27	0.08	0.09434	0.44899
Gln_4	2.33	**0.04**	0.59	0.25	0.02671	1.14662
Asp_3	-1.34	0.21	-0.09	0.07	-0.2349	0.05824
$GABA_4$	0.98	0.35	0.19	0.19	-0.23558	0.60891
Creatine	0.43	0.68	0.00	0.01	-0.02078	0.03078
Tau_1	-1.68	0.12	-0.02	0.01	-0.05809	0.00809
Myo_5	-2.28	**0.05**	-0.02	0.01	-0.0461	-<0.00156
Tau_2	-1.01	0.34	-0.01	0.01	-0.03216	0.01216
Myo_3	-1.14	0.28	-0.04	0.03	-0.10837	0.03503
Myo_4	-1.11	0.29	-0.01	0.01	-0.04011	0.01345
Glx_2	1.07	0.31	0.05	0.05	-0.0578	0.16447

Abbreviations:LactateC$_3$(Lac$_3$); AlanineC$_3$(AlaC$_3$); N-acetylaspartateC$_3$(NAA$_3$); γ-aminobutyric acidC$_2$(GABA$_2$); γ-aminobutyric acidC$_3$(GABA$_3$); γ-aminobutyric acidC$_4$(GABA$_4$); GlutamateC$_4$(Glu$_4$); GlutamineC$_4$(Gln$_4$); Glu$_2$+Gln$_2$(Glx$_2$); Glu$_3$+Gln$_3$(Glx$_3$); Aspartic acidC$_3$(Asp$_3$); TaurineC$_1$(Tau$_1$); TaurineC$_2$(Tau$_2$); Myo-InositolC$_3$(Myo$_3$); Myo-InositolC$_4$(Myo$_4$); Myo-InositolC$_5$(Myo$_5$). Bold indicated statistical significance.

Supplementary Table S3. The metabolite enrichment levels in the CTR and HIRI groups at ZT12 with [2-^{13}C]-acetate infusion.

Metabolites	T value	P value	Mean difference	Standard error difference	CI for the difference	
					lower limit	upper limit
Lac_3	-1.80	0.10	-0.10	0.06	-0.22746	0.02403
Ala_3	2.61	**0.03**	0.70	0.27	0.10363	1.30608
$GABA_3$	-0.38	0.71	-0.07	0.18	-0.47361	0.33476
NAA_3	0.35	0.73	0.03	0.09	-0.17398	0.2397
Glx_3	-2.74	**0.02**	-0.11	0.04	-0.19072	-0.01957
$GABA_2$	0.34	0.74	0.04	0.11	-0.20409	0.27666
Glu_4	0.21	0.84	0.02	0.09	-0.17684	0.21342
Gln_4	1.18	0.26	0.53	0.45	-0.46995	1.53738
Asp_3	-0.81	0.44	-0.07	0.09	-0.27987	0.13072
$GABA_4$	-0.21	0.83	-0.02	0.12	-0.28205	0.23234
Creatine	0.16	0.88	0.00	0.02	-0.04179	0.04808
Tau_1	0.87	0.41	0.01	0.01	-0.01927	0.04385
Myo_5	-2.90	**0.02**	-0.03	0.01	-0.04849	-0.00637
Tau_2	-1.72	0.12	-0.03	0.02	-0.06567	0.00853
Myo_3	-2.79	**0.02**	-0.07	0.03	-0.12746	-0.01425

Myo_4	-1.12	0.29	-0.02	0.02	-0.06216	0.02045
Glx_2	-0.21	0.84	-0.01	0.07	-0.16248	0.13448

Abbreviations:LactateC$_3$(Lac$_3$); AlanineC$_3$(AlaC$_3$); N-acetylaspartateC$_3$(NAA$_3$); γ-aminobutyric acidC$_2$(GABA$_2$); γ-aminobutyric acidC$_3$(GABA$_3$); γ-aminobutyric acidC$_4$(GABA$_4$); GlutamateC$_4$(Glu$_4$); GlutamineC$_4$(Gln$_4$); Glu$_2$+Gln$_2$(Glx$_2$); Glu$_3$+Gln$_3$(Glx$_3$); Aspartic acidC$_3$(Asp$_3$); TaurineC$_1$(Tau$_1$); TaurineC$_2$(Tau$_2$); Myo-InositolC$_3$(Myo$_3$); Myo-InositolC$_4$(Myo$_4$); Myo-InositolC$_5$(Myo$_5$). Bold indicated statistical significance.

Supplementary Table S4. Two-way ANOVA of HIRI and time(ZT0/ZT12) with [2-^{13}C]-acetate infusion

Metabolites	Sum of Squares	Mean Square	F	P value	Partial η2
Lac_3					
Intercept	2.175	2.175	213.249	<0.001	0.918
Time	0	0	0.027	0.87	0.001
HIRI	0.004	0.004	0.439	0.515	0.023
Time*HIRI	0.044	0.044	4.338	**0.051**	0.186
Ala_3					
Intercept	97.555	97.555	94.27	<0.001	0.832
Time	0.059	0.059	0.057	0.814	0.003
HIRI	1.517	1.517	1.465	0.241	0.072
Time*HIRI	0.325	0.325	0.314	0.582	0.016
$GABA_3$					
Intercept	28.272	28.272	338.446	<0.001	0.947
Time	0.011	0.011	0.134	0.718	0.007
HIRI	0.005	0.005	0.059	0.811	0.003
Time*HIRI	0.173	0.173	2.071	0.166	0.098
NAA_3					
Intercept	4.259	4.259	246.692	<0.001	0.928
Time	0.006	0.006	0.332	0.571	0.017
HIRI	0.006	0.006	0.368	0.551	0.019
Time*HIRI	0	0	0.023	0.881	0.001
Glx_3					

Intercept	7.494	7.494	990.846	<0.001	0.981
Time	0	0	0.032	0.86	0.002
HIRI	0.004	0.004	0.531	0.475	0.027
Time*HIRI	0.044	0.044	5.873	**0.026**	0.236
$GABA_2$					
Intercept	30.474	30.474	333.415	<0.001	0.946
Time	0.026	0.026	0.283	0.601	0.015
HIRI	0.101	0.101	1.1	0.307	0.055
Time*HIRI	0.047	0.047	0.514	0.482	0.026
Glu_4					
Intercept	15.049	15.049	696.937	<0.001	0.973
Time	0.022	0.022	1.023	0.325	0.051
HIRI	0.128	0.128	5.915	**0.025**	0.237
Time*HIRI	0.085	0.085	3.95	0.061	0.172
Gln_4					
Intercept	97.578	97.578	237.617	<0.001	0.926
Time	0.197	0.197	0.48	0.497	0.025
HIRI	1.743	1.743	4.245	0.053	0.183
Time*HIRI	0.007	0.007	0.016	0.899	0.001
Asp_3					
Intercept	2.829	2.829	143.963	<0.001	0.883
Time	0.005	0.005	0.239	0.631	0.012
HIRI	0.043	0.043	2.167	0.157	0.102
Time*HIRI	0	0	0.001	0.973	0

$GABA_4$					
Intercept	8.879	8.879	119.999	<0.001	0.863
Time	0.192	0.192	2.601	0.123	0.12
HIRI	0.057	0.057	0.77	0.391	0.039
Time*HIRI	0.043	0.043	0.582	0.455	0.03
Creatine					
Intercept	0.056	0.056	66.826	<0.001	0.779
Time	0.001	0.001	0.754	0.396	0.038
HIRI	0	0	0.101	0.755	0.005
Time*HIRI	0	0	0.009	0.924	0
Tau_1					
Intercept	0.014	0.014	21.402	<0.001	0.53
Time	0.002	0.002	2.678	0.118	0.124
HIRI	0	0	0.524	0.478	0.027
Time*HIRI	0.002	0.002	2.678	0.118	0.124
Myo_5					
Intercept	0.047	0.047	173.423	<0.001	0.901
Time	0	0	1.28	0.272	0.063
HIRI	0.004	0.004	15.399	**0.001**	0.448
Time*HIRI	0	0	0.307	0.586	0.016
Tau_2					
Intercept	0.071	0.071	123.449	<0.001	0.867
Time	0.002	0.002	3.328	0.084	0.149
HIRI	0.002	0.002	3.96	0.061	0.172

Time*HIRI	0.001	0.001	0.99	0.332	0.05
Myo_3					
Intercept	0.092	0.092	37.456	<0.001	0.663
Time	0.002	0.002	0.66	0.427	0.034
HIRI	0.019	0.019	7.878	**0.011**	0.293
Time*HIRI	0.003	0.003	1.076	0.313	0.054
Myo_4					
Intercept	0.02	0.02	28.37	<0.001	0.599
Time	0.001	0.001	1.161	0.295	0.058
HIRI	0.002	0.002	3.261	0.087	0.146
Time*HIRI	0	0	0.35	0.561	0.018
Glx_2					
Intercept	1.075	1.075	107.529	<0.001	0.85
Time	0.001	0.001	0.052	0.823	0.003
HIRI	0.001	0.001	0.063	0.805	0.003
Time*HIRI	0.01	0.01	1.048	0.319	0.052

Abbreviations:LactateC$_3$(Lac$_3$); AlanineC$_3$(AlaC$_3$); N-acetylaspartateC$_3$(NAA$_3$); γ-aminobutyric acidC$_2$(GABA$_2$); γ-aminobutyric acidC$_3$(GABA$_3$); γ-aminobutyric acidC$_4$(GABA$_4$); GlutamateC$_4$(Glu$_4$); GlutamineC$_4$(Gln$_4$); Glu$_2$+Gln$_2$(Glx$_2$); Glu$_3$+Gln$_3$(Glx$_3$); Aspartic acidC$_3$(Asp$_3$); TaurineC$_1$(Tau$_1$); TaurineC$_2$(Tau$_2$); Myo-InositolC$_3$(Myo$_3$); Myo-InositolC$_4$(Myo$_4$); Myo-InositolC$_5$(Myo$_5$). Bold indicated statistical significance.

Supplementary Table S5. The metabolite enrichment levels in the CTR group at ZT0 and ZT12 with [1-^{13}C]-glucose infusion.

Metabolites	T value	P value	Mean difference	Standard error difference	CI for the difference	
					lower limit	upper limit
Lac_3	1.54	0.15	0.31	0.20	-0.13953	0.76397
Ala_3	3.84	**<0.001**	0.72	0.19	0.30142	1.13364
NAA_3	2.06	0.07	0.09	0.04	-0.00699	0.18112
Glx_3	0.52	0.62	0.03	0.07	-0.11206	0.1796
$GABA_2$	-0.21	0.84	-0.04	0.20	-0.48832	0.40455
Glu_4	2.41	**0.04**	0.20	0.09	0.01525	0.39468
Gln_4	-2.43	**0.04**	-0.31	0.13	-0.59024	-0.02552
Asp_3	0.70	0.50	0.17	0.24	-0.37349	0.71309
$GABA_4$	1.70	0.12	0.22	0.13	-0.06631	0.49793
Creatine	2.38	**0.04**	0.03	0.01	0.00158	0.04868
Tau_1	-0.92	0.38	-0.01	0.01	-0.03578	0.01491
Myo_5	0.68	0.51	0.01	0.01	-0.01276	0.02409
Tau_2	2.77	**0.02**	0.06	0.02	0.01116	0.10235
Glx_2	-4.34	**<0.001**	-0.24	0.06	-0.3621	-0.1163

Abbreviations:LactateC$_3$(Lac$_3$); AlanineC$_3$(AlaC$_3$); N-acetylaspartateC$_3$(NAA$_3$); γ-aminobutyric acidC$_2$(GABA$_2$); γ-aminobutyric acidC$_3$(GABA$_3$); γ-aminobutyric acidC$_4$(GABA$_4$); GlutamateC$_4$(Glu$_4$); GlutamineC$_4$(Gln$_4$); Glu$_2$+Gln$_2$(Glx$_2$); Glu$_3$+Gln$_3$(Glx$_3$); Aspartic acidC$_3$(Asp$_3$); TaurineC$_1$(Tau$_1$); TaurineC$_2$(Tau$_2$); Myo-InositolC$_3$(Myo$_3$); Myo-InositolC$_4$(Myo$_4$); Myo-InositolC$_5$(Myo$_5$). Bold indicated statistical significance.

Supplementary Table S6. The metabolite enrichment levels in the CTR and HIRI groups at ZT0 with [1-^{13}C]-glucose infusion.

Metabolites	T value	P value	Mean difference	Standard error difference	CI for the difference	
					lower limit	upper limit
Lac_3	0.51	0.62	0.06	0.11	-0.19144	0.30455
Ala_3	-0.04	0.97	-0.01	0.28	-0.6258	0.60414
NAA_3	0.67	0.52	0.03	0.04	-0.06288	0.11699
Glx_3	0.10	0.92	0.01	0.09	-0.19202	0.21085
$GABA_2$	-2.65	**0.02**	-0.48	0.18	-0.87464	-0.07566
Glu_4	0.67	0.52	0.06	0.09	-0.1335	0.24872
Gln_4	-2.85	**0.02**	-0.43	0.15	-0.77161	-0.09434
Asp_3	-0.16	0.88	-0.06	0.39	-0.9291	0.80677
$GABA_4$	-0.05	0.96	-0.01	0.25	-0.57201	0.54554
Creatine	2.56	**0.03**	0.03	0.01	0.00346	0.04935
Tau_1	-0.73	0.48	-0.01	0.02	-0.05056	0.02563
Myo_5	-0.86	0.41	-0.02	0.02	-0.06845	0.03034
Tau_2	0.73	0.48	0.02	0.03	-0.03857	0.07607
Glx_2	-0.66	0.52	-0.08	0.12	-0.34246	0.18566

Abbreviations:LactateC_3(Lac_3); AlanineC_3(AlaC_3); N-acetylaspartateC_3(NAA_3); γ-aminobutyric acidC_2($GABA_2$); γ-aminobutyric acidC_3($GABA_3$); γ-aminobutyric acidC_4($GABA_4$); GlutamateC_4(Glu_4); GlutamineC_4(Gln_4); Glu_2+Gln_2(Glx_2); Glu_3+Gln_3(Glx_3); Aspartic acidC_3(Asp_3); TaurineC_1(Tau_1); TaurineC_2(Tau_2); Myo-InositolC_3(Myo_3); Myo-InositolC_4(Myo_4); Myo-InositolC_5(Myo_5). Bold indicated statistical significance.

Supplementary Table S7. The metabolite enrichment levels in the CTR and HIRI groups at ZT12 with [1-^{13}C]-glucose infusion.

Metabolites	T value	P value	Mean difference	Standard error difference	CI for the difference	
					lower limit	upper limit
Lac_3	0.02	0.98	0.01	0.22	-0.48324	0.49354
Ala_3	-6.07	**<0.001**	-1.08	0.18	-1.48338	-0.67805
NAA_3	-2.26	**0.05**	-0.11	0.05	-0.21935	<0.00114
Glx_3	-4.45	**<0.001**	-0.19	0.04	-0.27993	-0.09132
$GABA_2$	-2.58	**0.03**	-0.55	0.21	-1.03353	-0.06684
Glu_4	-6.04	**<0.001**	-0.41	0.07	-0.5571	-0.25352
Gln_4	-3.96	**<0.001**	-0.28	0.07	-0.44715	-0.12223
Asp_3	-1.19	0.26	-0.25	0.21	-0.72966	0.22668
$GABA_4$	-4.16	**<0.001**	-0.99	0.24	-1.52857	-0.45115
Creatine	-5.93	**<0.001**	-0.05	0.01	-0.07276	-0.03258
Tau_1	-0.57	0.58	-0.01	0.01	-0.03791	0.02269
Myo_5	1.44	0.18	0.02	0.01	-0.00931	0.04198
Tau_2	-2.68	**0.02**	-0.02	0.01	-0.03354	-0.00286
Glx_2	-0.41	0.69	-0.04	0.09	-0.24076	0.16669

Abbreviations:LactateC_3(Lac_3); AlanineC_3($AlaC_3$); N-acetylaspartateC_3(NAA_3); γ-aminobutyric acidC_2($GABA_2$); γ-aminobutyric acidC_3($GABA_3$); γ-aminobutyric acidC_4($GABA_4$); GlutamateC_4(Glu_4); GlutamineC_4(Gln_4); Glu_2+Gln_2(Glx_2); Glu_3+Gln_3(Glx_3); Aspartic acidC_3(Asp_3); TaurineC_1(Tau_1); TaurineC_2(Tau_2); Myo-InositolC_3(Myo_3); Myo-InositolC_4(Myo_4); Myo-InositolC_5(Myo_5). Bold indicated statistical significance.

Supplementary Table S8. Two-way ANOVA of HIRI and time(ZT0/ZT12) with [1-^{13}C]-glucose infusion

Metabolites	Sum of Squares	Mean Square	F	P value	Partial η2
Lac_3					
Intercept	97.067	97.067	1216.757	<0.001	0.985
Time	0.469	0.469	5.88	**0.025**	0.236
HIRI	0.005	0.005	0.068	0.797	0.004
Time*HIRI	0.004	0.004	0.047	0.83	0.002
Ala_3					
Intercept	221.47	221.47	1373.79	<0.001	0.986
Time	0.191	0.191	1.182	0.291	0.059
HIRI	1.702	1.702	10.558	**0.004**	0.357
Time*HIRI	1.635	1.635	10.143	**0.005**	0.348
NAA_3					
Intercept	1.589	1.589	283.099	<0.001	0.937
Time	0.002	0.002	0.357	0.557	0.018
HIRI	0.01	0.01	1.734	0.204	0.084
Time*HIRI	0.027	0.027	4.753	**0.042**	0.2
Glx_3					
Intercept	13.013	13.013	858.919	<0.001	0.978
Time	0.023	0.023	1.533	0.231	0.075
HIRI	0.044	0.044	2.928	0.103	0.134
Time*HIRI	0.054	0.054	3.587	0.074	0.159
$GABA_2$					

Intercept	105.285	105.285	959.451	<0.001	0.981
Time	0.036	0.036	0.328	0.573	0.017
HIRI	1.502	1.502	13.686	**0.002**	0.419
Time*HIRI	0.008	0.008	0.073	0.79	0.004
Glu$_4$					
Intercept	50.282	50.282	2884.328	<0.001	0.993
Time	0.004	0.004	0.23	0.637	0.012
HIRI	0.173	0.173	9.907	0.005	0.343
Time*HIRI	0.306	0.306	17.561	**<0.001**	0.48
Gln$_4$					
Intercept	39.055	39.055	905.434	<0.001	0.979
Time	0.312	0.312	7.238	0.014	0.276
HIRI	0.736	0.736	17.058	**0.001**	0.473
Time*HIRI	0.031	0.031	0.728	0.404	0.037
Asp$_3$					
Intercept	70.001	70.001	235.452	<0.001	0.925
Time	0.032	0.032	0.107	0.747	0.006
HIRI	0.14	0.14	0.47	0.501	0.024
Time*HIRI	0.052	0.052	0.174	0.681	0.009
GABA$_4$					
Intercept	37.265	37.265	215.95	<0.001	0.919
Time	0.424	0.424	2.459	0.133	0.115
HIRI	1.437	1.437	8.33	**0.009**	0.305
Time*HIRI	1.363	1.363	7.896	**0.011**	0.294

Creatine					
Intercept	0.148	0.148	549.106	<0.001	0.967
Time	0.001	0.001	4.406	**0.049**	0.188
HIRI	0.001	0.001	3.659	0.071	0.161
Time*HIRI	0.009	0.009	33.169	**<0.001**	0.636
Tau_1					
Intercept	0.045	0.045	64.927	<0.001	0.774
Time	0	0	0.528	0.476	0.027
HIRI	0.001	0.001	0.83	0.374	0.042
Time*HIRI	0	0	0.049	0.828	0.003
Myo_5					
Intercept	0.065	0.065	68.703	<0.001	0.783
Time	0.003	0.003	3.31	0.085	0.148
HIRI	0	0	0.011	0.917	0.001
Time*HIRI	0.002	0.002	1.9	0.184	0.091
Tau_2					
Intercept	0.135	0.135	122.141	<0.001	0.865
Time	0.008	0.008	7.581	**0.013**	0.285
HIRI	0	0	0	0.984	0
Time*HIRI	0.002	0.002	1.766	0.2	0.085
Residual	0.021	0.001			
Glx_2					
Intercept	5.968	5.968	182.775	<0.001	0.906
Time	0.273	0.273	8.356	**0.009**	0.305

HIRI	0.019	0.019	0.583	0.455	0.03
Time*HIRI	0.002	0.002	0.075	0.787	0.004

Abbreviations:LactateC$_3$(Lac$_3$); AlanineC$_3$(AlaC$_3$); N-acetylaspartateC$_3$(NAA$_3$); γ-aminobutyric acidC$_2$(GABA$_2$); γ-aminobutyric acidC$_3$(GABA$_3$); γ-aminobutyric acidC$_4$(GABA$_4$); GlutamateC$_4$(Glu$_4$); GlutamineC$_4$(Gln$_4$); Glu$_2$+Gln$_2$(Glx$_2$); Glu$_3$+Gln$_3$(Glx$_3$); Aspartic acidC$_3$(Asp$_3$); TaurineC$_1$(Tau$_1$); TaurineC$_2$(Tau$_2$); Myo-InositolC$_3$(Myo$_3$); Myo-InositolC$_4$(Myo$_4$); Myo-InositolC$_5$(Myo$_5$). Bold indicated statistical significance.

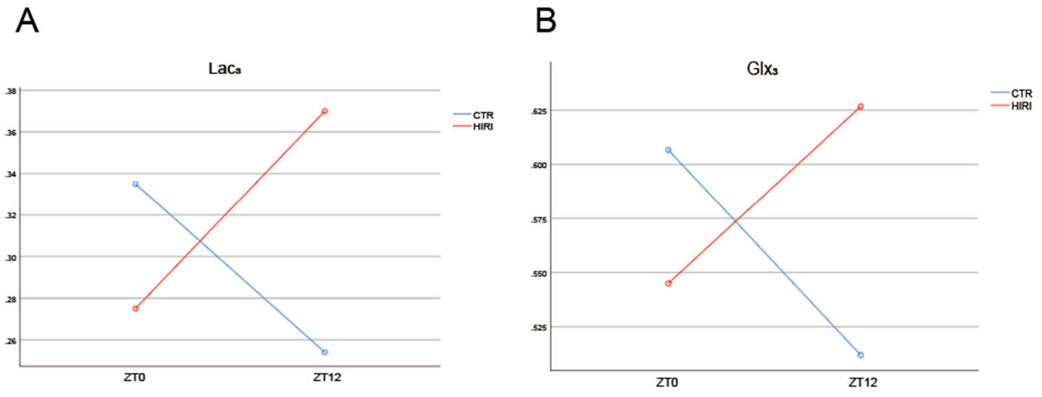

Supplementary Figure S1. Comparative Mean Values of Metabolites Lac_3 and $Glx_3$with [2-^{13}C]-acetate infusion。Abbreviations: LactateC_3(Lac_3); Glu_3+Gln_3(Glx_3).

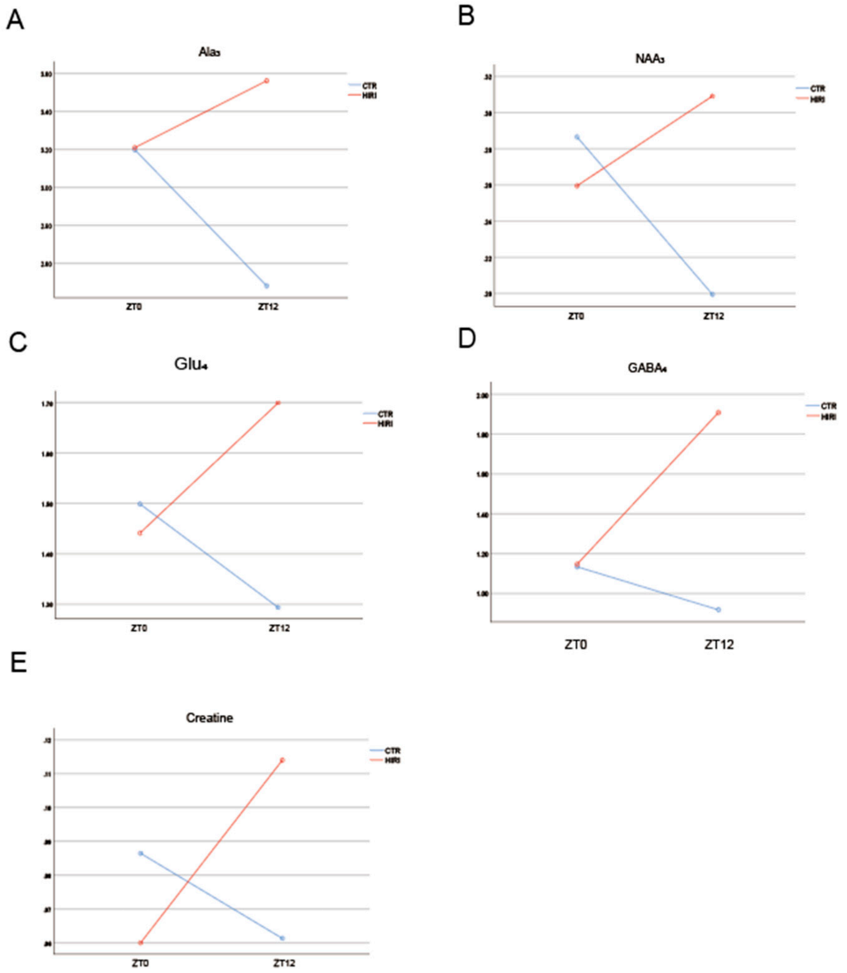

Supplementary Figure S2. Comparative Mean Values of Metabolites Ala_3 , NAA_3, Glu_4, $GABA_4$ and Creatine with[1-^{13}C]-glucose infusion. Abbreviations: AlanineC_3(AlaC_3); N-acetylaspartateC_3(NAA_3); γ-aminobutyric acidC_4($GABA_4$); GlutamateC_4(Glu_4).
